# Prevalence and proportion estimate of asymptomatic *Plasmodium* infection in Asia: a systematic review and meta-analysis

**DOI:** 10.1038/s41598-023-37439-9

**Published:** 2023-06-27

**Authors:** Manas Kotepui, Kwuntida Uthaisar Kotepui, Frederick Ramirez Masangkay, Aongart Mahittikorn, Polrat Wilairatana

**Affiliations:** 1 Medical Technology, School of Allied Health Sciences, Walailak University, Tha Sala, Nakhon Si Thammarat, Thailand; 2 Department of Medical Technology, University of Santo Tomas, Manila, Philippines; 3 Department of Protozoology, Faculty of Tropical Medicine, Mahidol University, Bangkok, Thailand; 4 Department of Clinical Tropical Medicine, Faculty of Tropical Medicine, Mahidol University, Bangkok, Thailand

**Keywords:** Infectious diseases, Malaria, Parasitic infection

## Abstract

Asymptomatic *Plasmodium* infection raises a problem for the persistent transmission of malaria in low-endemic areas such as Asia. This systematic review was undertaken to estimate the prevalence and proportion of asymptomatic *Plasmodium* infection in Asia. The systematic review was registered at PROSPERO (ID: CRD42022373664). The research followed the Preferred Reporting Items for Systematic Reviews and Meta-Analyses. A comprehensive search of five databases, Ovid, Scopus, MEDLINE, PubMed, and Embase, was conducted to identify studies of asymptomatic *Plasmodium* infection in Asian countries. The pooled prevalence of asymptomatic *Plasmodium* infection, the pooled proportion of asymptomatic *Plasmodium* infection among all parasitised individuals, and the associated 95% confidence intervals were estimated using a random-effects model. A total of 916 articles were retrieved, and 87 articles that met the criteria were included in the systematic review. The pooled prevalence of asymptomatic *Plasmodium* infection among enrolled participants in Southeast Asia, South Asia, and Western Asia was 5.8%, 9.4%, and 8.4%, respectively. The pooled proportion of asymptomatic *Plasmodium* infection among all parasitised individuals in Southeast Asia, South Asia, and Western Asia was 89.3%, 87.2%, and 64.8%, respectively. There was a low prevalence of asymptomatic *Plasmodium* infection, but there was a high proportion of asymptomatic *Plasmodium* infection per all parasitised individuals in different parts of Asia. These results may support and facilitate elimination and control programs for asymptomatic *Plasmodium* infection in Asia.

## Introduction

Asymptomatic *Plasmodium* infection is often characterised by very low parasitaemia, below the detection limit of light microscopic examination or malaria rapid diagnostic tests (RDTs), the current standard diagnoses. In addition, asymptomatic *Plasmodium* infection represents a hidden burden for communities as the clinical conversion from asymptomatic to symptomatic infection can occur^1^. Moreover, mosquitoes that suck blood from asymptomatic parasite carriers can transmit malaria to other people^2^. Nguitragool et al. showed that the risk factors for asymptomatic *Plasmodium* infection included male gender, participant age, feeling sick at the time of the survey, and place of residency^3^. Okell et al. showed that the proportion of sub-microscopic carriers was much higher in adults than in children^4^. By allowing persistent, low-level infections, partially effective antimalarial treatment may have contributed to the levels of sub-microscopic carriage^5^. Zhao et al. demonstrated that people who did not use bed nets or indoor residual spray, as well as those who lived in substandard homes or further distant from clinics, had increased odds of developing an asymptomatic infection^6^. Another factor was the persistence of submicroscopic *Plasmodium* infections after inadequate doses of antimalarials were used or drug resistance was present^7^. For instance, the treatment of *P. falciparum* or mixed species infections has been linked to an increased likelihood of *P. vivax* incidence^8,9^.

Malaria control measures rely heavily on the detection of asymptomatic *Plasmodium* infection. As malaria transmission has decreased in Asia, the high prevalence of asymptomatic and sub-microscopic infections has emerged as one of the greatest obstacles. Asymptomatic infected individuals do not typically seek treatment; consequently, parasites persist in these individuals, sustaining local transmission. It is widely accepted that asymptomatic *Plasmodium* carriers present a unique challenge for elimination programs as they provide a transmission reservoir capable of sustaining malaria endemicity^10,11^. Although blood film microscopy and RDTs are inexpensive and widely used in large-scale malaria surveys, they lack the sensitivity to detect infections in individuals who are asymptomatic and/or have sub-microscopic parasite densities. There is a need for more sensitive molecular tools, such as polymerase chain reaction (PCR), to support elimination strategies and target resources to areas of residual malaria transmission.

Based on WHO reports, the WHO South-East Asia Region comes in second to Africa in terms of malaria burden, with 2% of the burden of malaria cases globally. India was responsible for 83% of the cases in Asia, whereas Sri Lanka was declared malaria-free in 2016. In addition, malaria deaths decreased by 75%, from 2000 to 2020^12^. We conducted a systematic review to identify hotspots, describe the prevalence of asymptomatic *Plasmodium* infection among enrolled participants in the literature, and determine the proportion of asymptomatic *Plasmodium* infection among malaria-positive cases to maintain progress and eradicate malaria in the majority of Asia, as well as to understand the epidemiology of asymptomatic *Plasmodium* infection in Asia and the impact of malaria control efforts.

## Methods

### Protocol and registration

This systematic review was registered at PROSPERO (ID: CRD42022373664). The reports of the present systematic review and meta-analysis followed the Preferred Reporting Items for Systematic Reviews and Meta-Analyses (PRISMA) statement for reporting systematic reviews^13^.

### Research question

The research questions followed the PICo (Participant, Phenomena of Interest, Context) framework^14^. P stood for participants in the included studies, I stood for asymptomatic *Plasmodium* infections, and Co stood for Asian countries.

### Definition of asymptomatic malaria

Asymptomatic *Plasmodium* infection is defined as the presence of malaria parasitemia in an individual without fever or other signs and symptoms of malaria, according to RDTs, microscopy, or PCR at the time of the survey^15^.

### Outcomes

There were two outcomes of the systematic review. (1) the pooled prevalence of asymptomatic *Plasmodium* infection among enrolled participants in all studies and (2) the pooled proportion of asymptomatic *Plasmodium* infection among all malaria-positive cases.

### Search procedures and study inclusion

A comprehensive search of five databases (Ovid, Scopus, MEDLINE, PubMed, and Embase) was conducted to identify studies of asymptomatic *Plasmodium* infection in Asian countries (inception to October 16, 2022). The search strategy includes the terms "malaria OR Plasmodium OR "remittent fever" OR "marsh fever" OR paludism) AND (“asymptomatic infection” OR inapparent OR subclinical OR presymptomatic) AND (Asia OR Asian)". Details of the search strategy in five databases can be found in Table S1. We restricted the search to studies published after 2000. We included cross-sectional studies and cohort studies (at baseline) of asymptomatic *Plasmodium* infection among participants in Asian countries. Non-research articles, case reports, case series, case–control studies, conference abstracts, and in vitro and in vivo studies were excluded from this study. Two authors (MK and KUK) performed the study selection and data extraction and assessed the risk of bias among the included studies. Disagreement between the two authors was resolved by another author (AM).

The systematic review and meta-analysis were reported following the PRISMA checklist^13^. The risk of bias was assessed using Strengthening the Reporting of Observational Studies in Epidemiology (STROBE) for cross-sectional and cohort studies^16^. There were scale ratings to categorise the quality of studies as good, fair, or poor in cases where the percentile of the total score (overall, 22 items and scores) were more than 75%, 50–74%, or less than 50%, respectively^17,18^.

### Statistical analysis

(1) The pooled prevalence of asymptomatic *Plasmodium* infection among enrolled participants in all studies, and (2) the pooled proportion of asymptomatic *Plasmodium* infection among all malaria-positive cases and associated 95% confidence intervals (CIs) were estimated using a random-effects model (DerSimonian and Laird method)^19^. The meta-regression analysis was performed to determine whether covariates affect the prevalence estimate or not. The subgroup analyses of the publication year, parts of the continent, participants' group, age group, and method for malaria detection were performed to explore the source of heterogeneity and demonstrate the differences in prevalence in specific groups of participants. The heterogeneity of the effect estimates was assessed using the Chi-square and I^2^ statistics as described previously^20^. Publication bias was not assessed across all included studies because there is no clear definition or consensus regarding what constitutes a positive result in a proportional meta-analysis^21^.

## Results

### Search results

**Figure 1:**
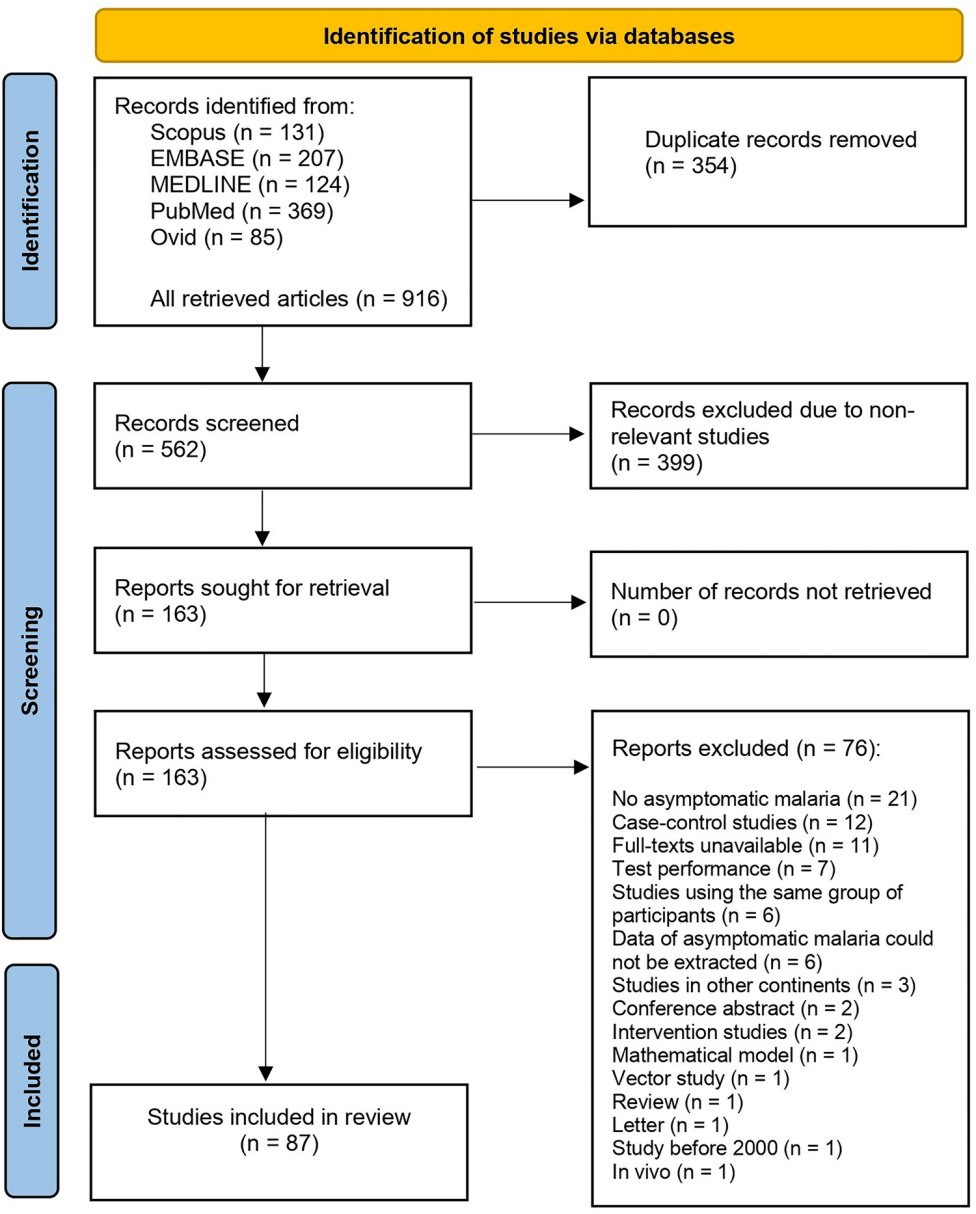
The study flow diagram illustrating the progression of the study selection process.

A total of 916 articles were retrieved from database searches (207 from Embase, 124 from MEDLINE, 85 from Ovid, 369 from PubMed, and 131 from Scopus). The 354 duplicates were removed, and 562 remained for the title and abstract screening. After screening 562 articles, we excluded 399 that did not relate to the participants and outcomes of interest. Subsequently, 163 relevant articles were included in the full-text examination. After 163 articles were examined for full-texts, 76 articles were excluded with specific reasons, and 87 articles^3,6,7,10,11,22–103^ that met the criteria were included in the systematic review (Fig. 1).

### Characteristics of the included studies

All details about the characteristics of the included studies are shown in Table S2. Briefly, most of the included studies were conducted in Southeast Asia (60 studies, 69%), followed by South Asia (20 studies, 23%), and Western Asia (7 studies, 8.05%). In Southeast Asia (60 studies), most of the studies were conducted in Thailand (10 studies, 16.7%), Myanmar (9 studies, 15%), Cambodia (8 studies, 13.3%), Malaysia (8 studies, 13.3%), Lao PDR (7 studies, 11.7%), Indonesia (6 studies, 10%), Vietnam (6 studies, 10%), the Philippines (2 studies, 3.33%), the Democratic Republic of East Timor (1 study, 1.67%), and three studies were conducted in multi-sites (1 in the Thailand-Myanmar border/Cambodia, 1 in the Thailand–Myanmar border/Cambodia/Vietnam, 1 in Thailand and Myanmar). In South Asia (20 studies), most of the studies were conducted in India (14 studies, 70%), followed by Bangladesh (4 studies, 20%), Pakistan (1 study, 5%), and Bhutan (1 study, 5%). In Western Asia (7 studies), most of the studies were conducted in Yemen (4 studies, 57.1%), followed by Iran (2 studies, 28.6%), and Saudi Arabia (1 study, 14.3%) (Table 1). Most of the included studies enrolled participants in communities (67 studies, 77%), followed by children (6 studies, 6.9%), pregnant women (6 studies, 6.9%), migrant workers (5 studies, 5.75%), participants who had undergone training in the forest (2 studies, 2.3%), participants in communities, and migrant workers (1 study, 1.2%). Most of the included studies used a combination of microscopy, RDT, and PCR for the detection of *Plasmodium* (26 studies, 29.9%), followed by a combination of microscopy and PCR (24 studies, 27.6%); microscopy alone (10 studies, 11.5%); RDT and PCR (10 studies, 11.5%); PCR alone (8 studies, 9.2%); microscopy and RDT (4 studies, 4.6%); RDT alone (3 studies, 3.45%); LAMP and PCR (1 study, 1.2%); and one study used a combination of microscopy, LAMP, PCR, restriction enzyme digestion, and DNA sequencing (1 study, 1.2%) (Table 1). Overall, there were 12,316 asymptomatic *Plasmodium* infections among 302,772 participants.

**Table 1:** Prevalence of asymptomatic *Plasmodium* infection in Asian countries.

		Pooled prevalence (%)	95% CI (%)	I^2^	Number of studies
**Overall prevalence**	**Overall**	5.0	5.0–6.0	99.37	87
	Molecular methods (combined)	6.3	5.8–6.7	93.39	70
	Non-molecular methods	3.4	2.8–3.9	99.31	17
**Parts of Asia**					
**Southeast Asia**	**Overall **	5.6	5.1–6.1	99.39	60
	**Molecular methods** (combined)	5.8	5.3–6.3	99.38	56
	Microscopy/RDT/PCR	7.0	4.8–9.1	ND	15
	RDT/PCR	4.0	3.0–4.9	ND	9
	Microscopy/PCR	6.1	5.2–7.0	ND	22
	PCR	7.6	5.2–9.9	ND	8
	Microscopy/LAMP/PCR and restriction enzyme digestion/DNA sequencing	2.1	1.7–2.6	ND	1
	LAMP/PCR	0.3	0.1–0.7	ND	1
	**Non-molecular methods**	2.6	0.3–5.0	98.84	4
	Microscopy	1.8	1.6–2.0	ND	3
	Microscopy/RDT	5.6	5.2–0.6	ND	1
	**Indonesia**	11.6	8.3–14.8	99.68	6
	Molecular methods (combined)	11.6	8.3–14.8	99.68	6
	Microscopy/RDT/PCR	6.50	5.10–8.30	ND	1
	Microscopy/PCR	19.0	0–47.80	ND	3
	LAMP/PCR	0.30	0.10–0.70	ND	1
	PCR	11.9	8.2–17.0	ND	1
	**Myanmar**	9.2	5.3–13.1	99.45	9
	Molecular methods (combined)	9.2	5.3–13.1	99.45	9
	RDT/PCR	0.60	0.50–0.80	ND	1
	Microscopy/PCR	9.9	0.30–19.6	ND	3
	Microscopy/RDT/PCR	10.6	2.90–18.2	99.36	5
	**Lao PDR**	7.0	3.0–10.9	97.94	7
	Molecular methods (combined)	7.0	3.0–10.9	97.94	7
	RDT/PCR	8.90	0–20.1	ND	3
	Microscopy/RDT/PCR	4.80	3.80–5.80	ND	3
	Microscopy/PCR	7.9	5.5–11.2	ND	1
	**Cambodia**	5.9	3.5–8.2	99.33	8
	Molecular methods (combined)	5.9	3.1–8.7	99.40	7
	RDT/PCR	5.2	4.5–5.8	ND	2
	Microscopy/RDT/PCR	6.2	1.1–11.3	ND	3
	PCR	4.6	4.3–4.9	ND	2
	Non-molecular methods	5.6	5.2–6.0	ND	1
	Microscopy/RDT	5.6	5.2–6.0	ND	1
	**Malaysia**	2.5	1.8–3.3	97.54	8
	Molecular methods (combined)	2.5	1.8–3.3	97.54	8
	PCR	0.9	0.2–1.5	ND	3
	Microscopy/PCR	6.4	2.3–10.5	97.85	5
	**Thailand**	2.4	1.5–3.2	96.56	10
	Molecular methods (combined)	3.0	1.6–4.4	97.02	7
	Microscopy/PCR	4.3	2.0–6.6	86.67	4
	RDT/PCR	0.6	0.5–0.8	ND	2
	Microscopy/LAMP/PCR	2.1	1.7–2.6	ND	1
	Non-molecular methods	1.8	1.6–2.0	ND	3
	Microscopy	1.8	1.6–2.0	ND	3
	**Vietnam**	2.4	1.0–3.7	96.33	6
	Molecular methods (combined)	2.4	1.0–3.7	96.33	6
	Microscopy/PCR	2.0	0–0.41	ND	3
	RDT/PCR	1.7	1.3–2.2	ND	1
	PCR	5.6	2.6–11.6	ND	1
	Microscopy/RDT/PCR	3.1	2.3–4.2	ND	1
	**Philippines**	2.0	1.0–3.0	ND	2
	Molecular methods (combined)	2.0	1.0–3.0	ND	2
	Microscopy/RDT/PCR	0.3	0.2–0.6	ND	1
	Microscopy/PCR	0.1	0–0.4	ND	1
	**Democratic Republic of East Timor** (Microscopy/PCR)	5.1	2.9–8.9	ND	1
	**Thailand–Myanmar border, Cambodia** (PCR)	27.5	26.2–28.8	ND	1
	**Thailand and Myanmar** (Microscopy/PCR)	4.6	3.7–5.7	ND	1
	**Thailand–Myanmar border, Cambodia, Vietnam** (Microscopy/RDT/PCR)	9.1	8.3–9.9	ND	1
**Western Asia**	**Overall**	7.6	5.0–10.1	98.19	7
	**Molecular methods** (combined)	8.4	0–17.3	ND	3
	Microscopy/RDT/PCR	8.4	0–17.3	ND	2
	Microscopy/PCR	0.2	0.1–0.3	ND	1
	**Non-molecular methods**	7.8	1.4–14.3	97.86	4
	Microscopy	7.8	1.4–14.3	97.86	4
	**Yemen**	13.3	7.9–18.7	93.29	4
	Molecular methods (combined)	23.0	19.4–26.9	ND	1
	Microscopy/RDT/PCR	23.0	19.4–26.9	ND	1
	Non-molecular methods	10.2	7.8–12.5	ND	3
	Microscopy	10.2	7.8–12.5	ND	3
	**Saudi Arabia** (Microscopy/RDT/PCR)	2.9	1.5–5.6	ND	1
	**Iran**	0.2	0.1–0.3	ND	2
	Molecular methods (combined)	0.2	0.1–0.3	ND	1
	Microscopy/PCR	0.2	0.1–0.3	ND	1
	Non-molecular methods	0.7	0.4–1.5	ND	1
	Microscopy	0.7	0.4–1.5	ND	1
**South Asia**	**Overall **	5.7	5.0–6.3	99.47	20
	**Molecular methods** (combined)	9.4	6.5–12.2	99.40	11
	Microscopy/PCR	24.5	22.8–26.4	ND	1
	Microscopy/RDT/PCR	8.6	5.9–11.4	99.0	9
	RDT/PCR	0.3	0.1–0.8	ND	1
	**Non-molecular methods**	2.8	2.2–3.4	99.34	9
	Microscopy	5.0	2.9–7.1	ND	3
	Microscopy/RDT	6.4	2.4–10.4	ND	3
	RDT	1.5	0.9–2.2	ND	3
	**Bangladesh**	12.7	5.2–20.3	99.74	4
	Molecular methods (combined)	17.0	0.3–33.6	ND	3
	Microscopy/PCR	24.5	22.8–26.4	ND	1
	Microscopy/RDT/PCR	4.6	3.9–5.2	ND	2
	Non-molecular methods	0.3	0.1–0.5	ND	1
	Microscopy/RDT	0.3	0.1–0.5	ND	1
	**India**	4.6	3.9–5.2	99.45	14
	Molecular methods (combined)	7.3	4.3–10.3	98.94	6
	Microscopy/RDT/PCR	7.3	4.3–10.3	98.94	6
	Non-molecular methods	3.3	2.7–4.0	99.43	8
	RDT	1.5	0.9–2.2	ND	3
	Microscopy	5.0	2.9–7.1	ND	3
	Microscopy/RDT	4.1	3.3–4.9	ND	2
	**Pakistan** (Microscopy/RDT/PCR)	8.0	5.7–11.1	ND	1
	**Bhutan** (RDT/PCR)	0.3	0.1–0.8	ND	1
**Participants**	Participants in communities (all age groups)	5.9	5.5–6.3	99.49	60
	Molecular methods (combined)	6.3	5.8–6.8	99.47	54
	PCR	7.3	4.7–9.5	99.85	6
	RDT/PCR	2.5	1.7–3.3	98.31	7
	LAMP/PCR	0.3	0.1–0.7	ND	1
	Microscopy/PCR	6.9	6.0–7.7	99.44	19
	Microscopy/RDT/PCR	8.4	6.5–10.2	99.18	20
	Microscopy/LAMP/PCR and restriction enzyme digestion/DNA sequencing	2.1	1.7–2.6	ND	1
	Non-molecular methods	7.0	3.9–10.0	99.65	6
	Microscopy	7.9	6.4–9.5	ND	2
	RDT	0.1	0.1–0.1	ND	2
	Microscopy/RDT	5.7	5.3–6.1	ND	2
	Participants in communities (children)	7.9	6.4–9.4	88.70	8
	Molecular methods (combined)	8.0	5.4-–10.6	89.65	5
	PCR	11.9	8.2–17.0	ND	1
	RDT/PCR	6.3	4.1–9.5	ND	1
	Microscopy/PCR	4.5	3.3–5.7	ND	2
	Microscopy/RDT/PCR	7.5	6.9–8.0	ND	1
	Non-molecular methods	8.8	5.0–12.7	ND	3
	Microscopy	8.8	5.0–12.7	ND	3
	Participants in communities (adults)	8.3	3.0–13.6	98.29	4
	Molecular methods (combined)	10.2	1.9–18.5	ND	3
	RDT/PCR	19.7	17.2–22.5	ND	1
	Microscopy/RDT/PCR	3.6	2.9–4.2	ND	2
	Non-molecular methods	3.0	1.9–4.7	ND	1
	Microscopy	3.0	1.9–4.7	ND	1
	Pregnant women	2.2	1.4–3.1	98.65	6
	Molecular methods (combined)	6.3	4.9–7.7	ND	2
	Microscopy/RDT/PCR	6.3	4.9–7.7	ND	2
	Non-molecular methods	1.4	0.5–2.2	99.0	4
	Microscopy	1.8	1.6–2.0	ND	1
	RDT	0.2	0.1–0.3	ND	1
	Microscopy/RDT	0.4	0.2–0.6	ND	2
	Migrant workers	2.1	0.8–3.4	77.57	5
	Molecular methods (combined)	6.0	3.5–8.5	ND	2
	PCR	5.6	2.6–11.6	ND	1
	Microscopy/PCR	6.2	3.8–10.0	ND	1
	Non-molecular methods	0.1	0.5–1.7	ND	3
	Microscopy	0.1	0.5–1.7	ND	3
	Participants had undergone training in the forest (Microscopy/PCR)	0.7	0.1–1.3	ND	2
	Participants in communities (not specified age group) (Microscopy/RDT/PCR)	8.0	5.7–11.1	ND	1
	Participants in communities and migrant workers (RDT/PCR)	0.3	0.1–0.8	ND	1

*CI* confidence interval, *LAMP* Loop-mediated isothermal amplification, *ND* not determined, *PCR* polymerase chain reaction, *RDT* rapid diagnostic test.

### Quality of the included studies (risk of bias)

The quality of the included studies was demonstrated in Table S3. For the overall quality, all 87 studies were high-quality studies (100%). In terms of methodology quality, 86 studies^3,6,7,10,11,22–90,92–103^ were of high quality, while one^91^ was of moderate quality. All studies were included in the meta-analysis.

### Prevalence of asymptomatic *Plasmodium* infection among enrolled participants in Asia

**Figure 2:**
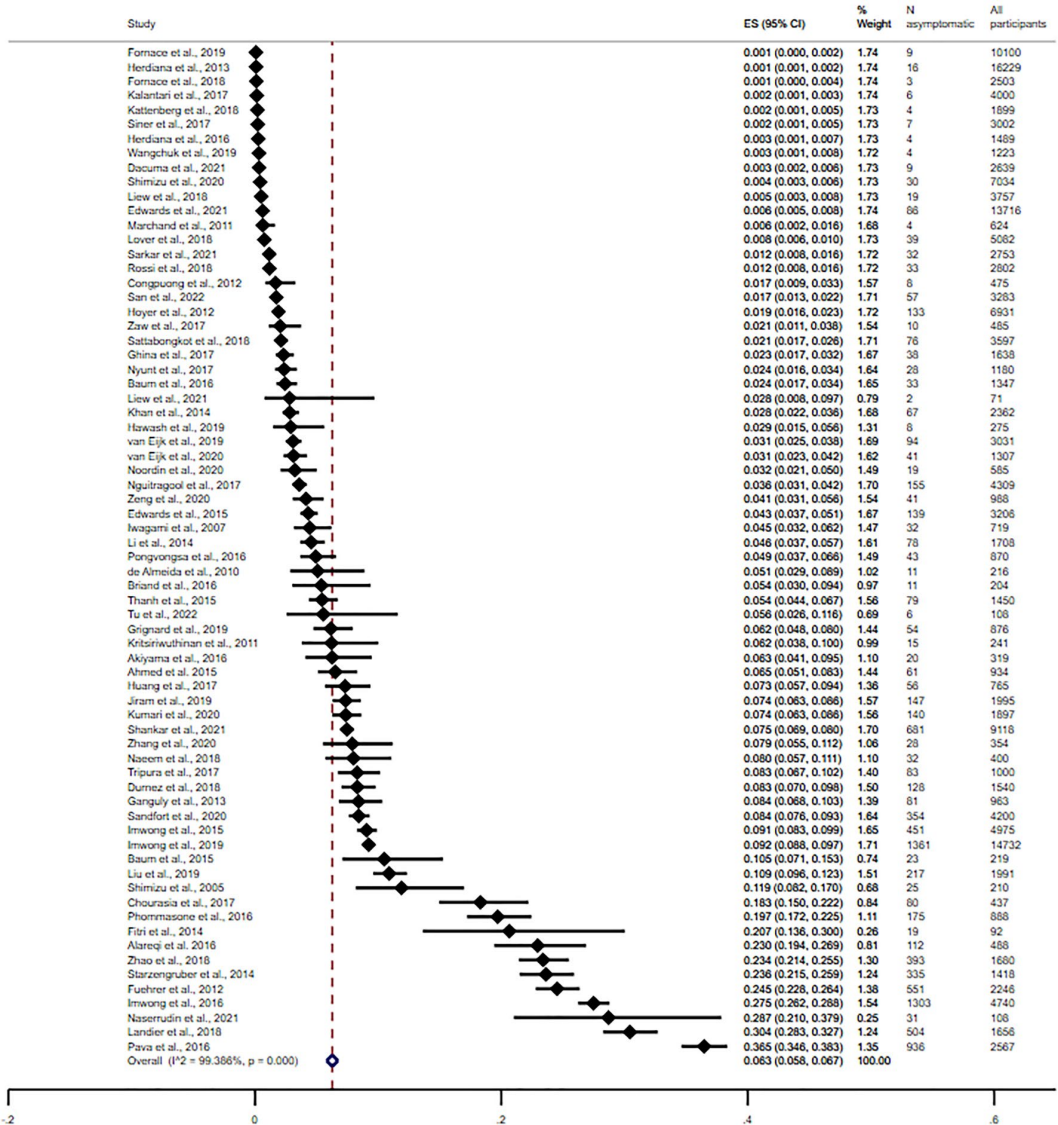
The pooled prevalence of asymptomatic *Plasmodium* infection among enrolled participants was 6.3% (95% CI 5.8–6.7%, I^2^: 93.39%, 70 studies) when the molecular method was used alone or in combination with other methods. Abbreviations: ES, prevalence estimate (× 100); CI, confidence interval (× 100).

**Figure 3:**
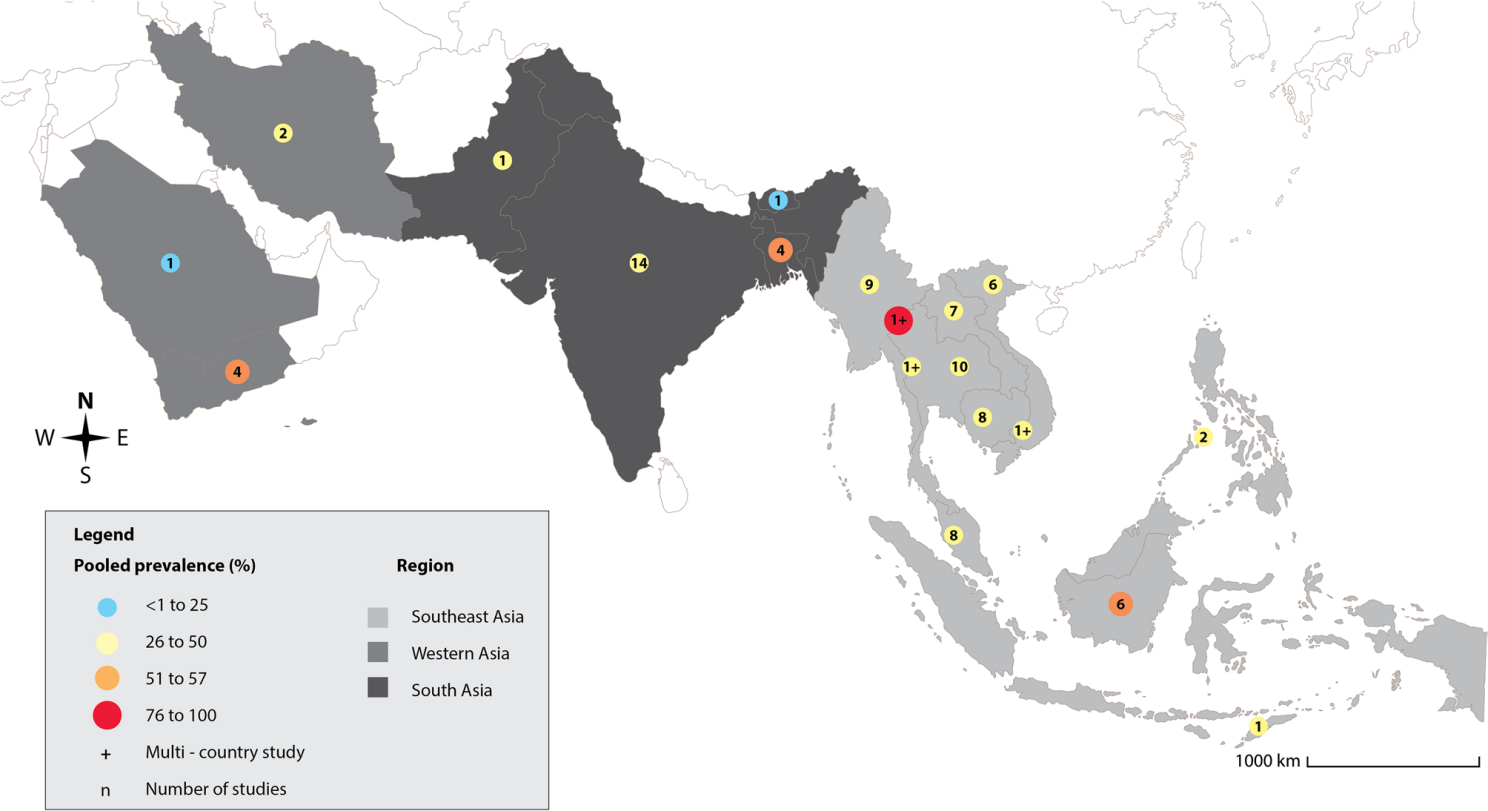
Geographic distribution and the pooled prevalence of asymptomatic *Plasmodium* infection in Asian countries. Southeast Asia: 5.6 and 60 Pooled prevalence and number of studies, respectively; Western Asia: 7.6 and 7 Pooled prevalence and number of studies, respectively; South Asia: 5.7 and 20 Pooled prevalence and number of studies, respectively. The figure was created by the Illustrator version CC 2023. The base map was from https://www.freepik.com/.

The pooled prevalence of asymptomatic *Plasmodium* infection among enrolled participants when the molecular method was used alone or in combination with other methods was 6.3% (95% CI 5.8–6.7%, I^2^: 93.39%, 70 studies, Fig. 2). Figure 3 depicts the geographical distribution and estimated prevalence of asymptomatic *Plasmodium* infection among participants enrolled in the included studies. The meta-regression analysis showed that none of the covariates, including the publication year, part of the Asian continent, country, participants' group, or method for malaria detection, significantly affected the prevalence estimate. Nevertheless, the meta-regression analysis showed that the country was the most-fitted covariate to explain the heterogeneity of the prevalence estimate (adjusted R-squared = 9.53%) (Table S4). Subgroup analyses of parts of the Asian continent, country, and participants' group were performed using data from studies that used the molecular method alone or in combination with other methods.

The subgroup analysis of parts of the Asian continent showed that the prevalence of asymptomatic *Plasmodium* infection among enrolled participants was 5.8% in Southeast Asia (95% CI 5.3–6.3%, I^2^: 99.38%, 56 studies), 9.4% in South Asia (95% CI 6.5–12.2%, I^2^: 99.40%, 11 studies), and 8.4% in Western Asia (95% CI 0–17.3%, 3 studies). The subgroup analysis of the country showed that the highest prevalence of asymptomatic *Plasmodium* infection among enrolled participants in Southeast Asia was found in a study conducted in the Thailand-Myanmar border and Cambodia (27.25%, 95% CI 26.2–28.8%)^55^, followed by Indonesia (11.6%, 95% CI 8.3–14.8%, I^2^: 99.68, 6 studies), and Myanmar (9.2%, 95% CI 5.3–11.3%, I^2^: 99.45%, 9 studies). The highest prevalence of asymptomatic *Plasmodium* infection among enrolled participants in Western Asia was found in Yemen (23.3%, 95% CI 19.4–26.9%)^24^, followed by Saudi Arabia (2.9%, 95% CI 1.5–5.6%)^49^, and Iran (0.2%, 95% CI 0.1–0.3%)^59^. The highest prevalence of asymptomatic *Plasmodium* infection among enrolled participants in South Asia was found in Bangladesh (17.0%, 95% CI: 0.3–33.6%, 3 studies), followed by Pakistan (8.0%, 95% CI 5.7–11.1%)^74^, India (7.3%, 95% CI 4.3%–10.3%, I^2^: 98.94%, 6 studies), and Bhutan (0.3%, 95% CI 0.1–0.8%)^100^. The subgroup analysis of participants showed that the highest prevalence of asymptomatic *Plasmodium* infection was among enrolled adults (10.2%, 95% CI 1.9–18.5%, 3 studies), followed by children (8.0%, 95% CI 5.4–10.6%, I^2^: 89.65%, 5 studies), participants in communities without age information (8.0%, 95% CI 5.7–11.1%)^74^, participants in communities with all age groups (6.3%, 95% CI 5.8–6.8%, I^2^: 99.47%, 54 studies), pregnant women (6.3%, 95% CI 4.9–7.7%, 2 studies), and migrant workers (6.0%, 95% CI 3.5–8.5%, 2 studies). Table 1 shows the prevalence of asymptomatic *Plasmodium* infection among enrolled participants in different parts of Asia, countries, and participants’ groups when molecular and non-molecular methods were used.

### Proportion of asymptomatic *Plasmodium* infections per parasitised individuals in Asia

**Figure 4:**
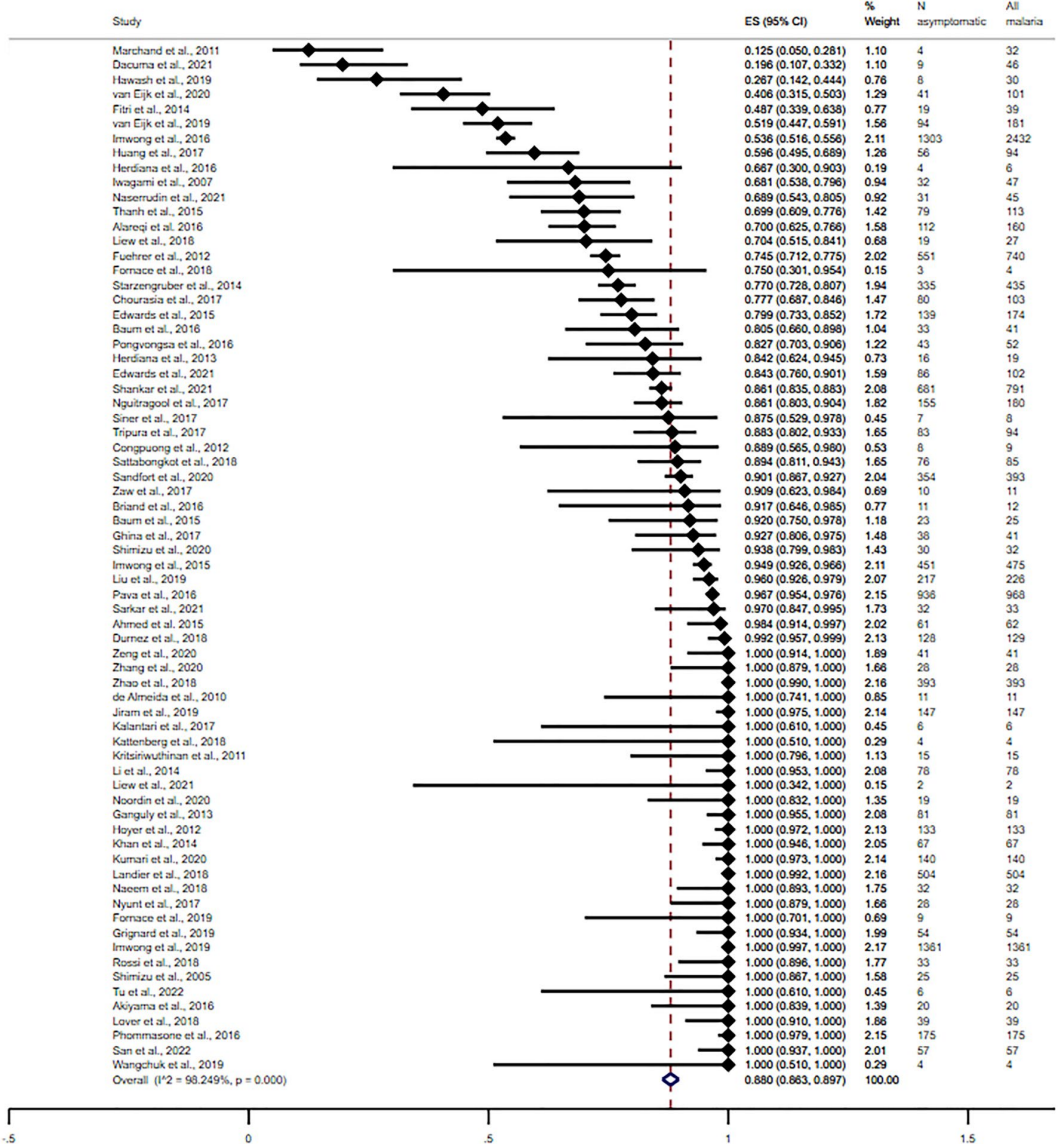
The pooled proportion of asymptomatic *Plasmodium* infection among all parasitised individuals was 88.0% (95% CI 86.3–89.7%, I^2^: 98.25%, 70 studies) when the molecular method was used alone or in combination with other methods. *ES* proportion estimate (× 100), *CI* confidence interval (× 100).

**Figure 5:**
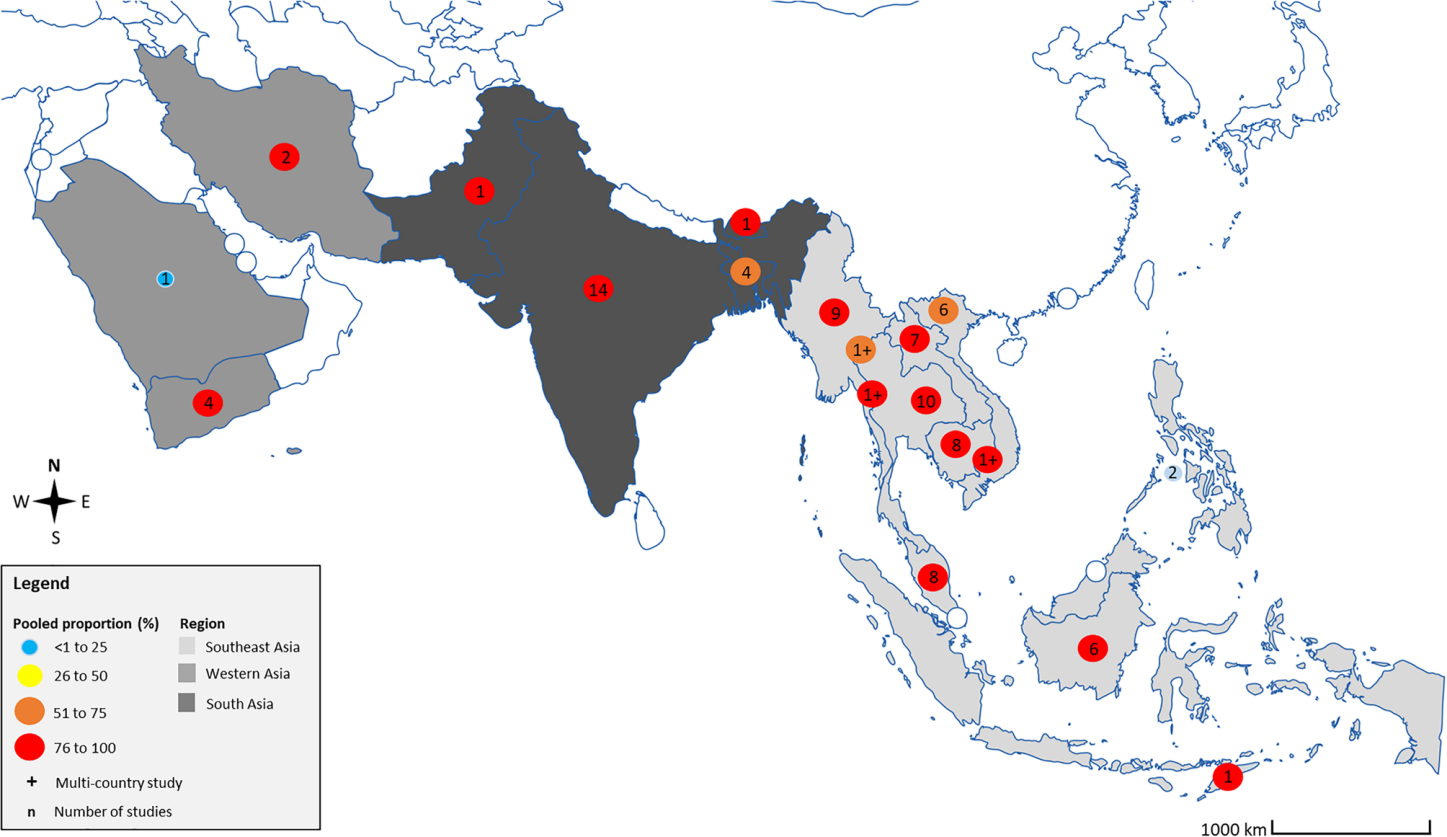
Geographic distribution and the pooled proportion of asymptomatic *Plasmodium* infection per all parasitised individuals in Asian countries. Southeast Asia: 87.6 and 60 Pooled proportion and number of studies respectively; Western Asia: 74.3 and 7 Pooled proportion and number of studies, respectively; South Asia: 78 and 20 Pooled proportion and number of studies, respectively. The figure was created by the Illustrator version CC 2023. The base map was from https://www.freepik.com/.

The pooled proportion of asymptomatic *Plasmodium* infection among all parasitised individuals when the molecular method was used alone or in combination with other methods was 88.0% (95% CI 86.3–89.7%, I^2^: 98.25%, 70 studies, Fig. 4). Figure 5 depicts the geographical distribution as well as the estimated proportion of asymptomatic *Plasmodium* infection cases among all parasitised individuals. The meta-regression analysis showed that the country and participants' group affected the proportion estimate, with a *P* value less than 0.01, but the publication year, being part of the Asian continent, or method for malaria detection did not affect the proportion estimate (Table S4). In addition, the meta-regression analysis showed that the participants' group and country were the most-fitted covariates to explain the heterogeneity of the proportion estimate (adjusted R-squared = 30.76% and 29.19%, respectively). Subgroup analyses of parts of the Asian continent, country, and participants' group were performed using data from studies that used the molecular method alone or in combination with other methods. The subgroup analysis of parts of the Asian continent showed that the highest proportion of asymptomatic *Plasmodium* infection per parasitised individuals was found in Southeast Asia (89.3%, 95% CI 84.7–91.1%, I^2^: 98.23%, 56 studies), followed by South Asia (87.2%, 95% CI 79.9–94.5%, I^2^: 98.10%, 11 studies), and Western Asia (64.8%, 95% CI 30.6–99.0%, 3 studies) when the molecular method was used alone or in combination with other methods (Table 2). The subgroup analysis of the country showed that the highest proportion of asymptomatic *Plasmodium* infection per parasitised individuals in Southeast Asia was found in Thailand/Myanmar (100%, 95% CI 93.5–100%)^67^, followed by the Democratic Republic of East Timor (100%, 95% CI 74.1–100%)^37^, Myanmar (96.5%, 95% CI 49.5–98.5%, I^2^: 91.64%, 9 studies), Lao PDR (94.1%, 95% CI 88.6–99.7%, I^2^: 82.04%, 7 studies), Cambodia (95.2%, 95% CI 92.4–98.0%, I^2^: 94.00%, 7 studies), Malaysia (93.6%, 95% CI 87.7–99.4%, I^2^: 78.37%, 7 studies), Indonesia (90.3%, 95% CI 83.6–97.1, I^2^: 88.25%, 6 studies), Thailand (89.7%, 95% CI 85.7–93.7%, I^2^: 28.58%, 7 studies), Vietnam (69.7%, 95% CI 38.4–100, I^2^: 98.48, 6 studies), and the Philippines (23.3%, 95% CI 12.3–34.4%, 2 studies). The highest proportion of asymptomatic *Plasmodium* infection per parasitised individuals in Western Asia was found in Iran (100%, 95% CI 61.1–100%)^59^, followed by Yemen (70.0%, 95% CI 62.5–76.6%)^24^, and Saudi Arabia (26.7%, 95% CI 14.2–44.4%). The highest proportion of asymptomatic *Plasmodium* infection per parasitised individuals in South Asia was found in Pakistan (100%, 95% CI 82.3–100%)^74^, followed by Bhutan (100%, 95% CI 51.0–100%)^100^, India (86.0%, 95% CI 77.1–95.0%, I^2^: 98.14, 6 studies), and Bangladesh (83.9%, 95% CI 66.6–100%, 3 studies). The subgroup analysis of participants showed that the highest proportion of asymptomatic *Plasmodium* infection per parasitised individuals was found in studies that enrolled participants in communities without age information (100%, 95% CI 89.3–100%)^74^, participants that had undergone training in the forest (100%, 95% CI 89.3–100%, 2 studies), and participants in communities/migrant workers (100%, 95% CI 51.0–100%)^100^. Table 2 shows the proportion of asymptomatic *Plasmodium* infection per parasitised individual in different parts of Asia, countries, and participants’ groups when molecular and non-molecular methods were used.

**Table 2:** Proportion of asymptomatic *Plasmodium* infection per all parasitised individuals in Asian countries.

		Pooled proportion (%)	95% CI (%)	I^2^	Number of studies
**Overall proportion**	**Overall**	84.4	82.6–86.3	98.61	87
	Molecular methods (combined)	88.0	86.3–89.7	98.25	70
	Non-molecular methods	72.2	61.9–82.5	98.23	17
Parts of Asia					
**Southeast Asia**	**Overall**	87.6	85.6–89.5	98.58	60
	**Molecular methods** (combined)	89.3	87.4–91.1	98.23	56
	Microscopy/RDT/PCR	83.9	79.5–88.3	97.27	15
	RDT/PCR	94.3	90.8–97.8	90.72	9
	Microscopy/PCR	88.6	85.2–91.9	94.56	22
	PCR	92.5	73.7–100	99.67	8
	Microscopy/LAMP/PCR and restriction enzyme digestion/DNA sequencing	89.4	81.1–94.3	ND	1
	LAMP/PCR	66.7	30.0–90.3	ND	1
	**Non-molecular methods**	66.1	51.9–80.4	97.71	4
	Microscopy	81.8	40.7–100	ND	3
	Microscopy/RDT	69.7	66.6–62.6	ND	1
	**Myanmar**	96.5	49.5–98.5	91.64	9
	Molecular methods (combined)	96.5	49.5–98.5	91.64	9
	RDT/PCR	84.3	76.0–90.1	ND	1
	Microscopy/PCR	99.3	96.7–100	ND	3
	Microscopy/RDT/PCR	91.2	84.2–98.1	94.57	5
	**Lao PDR**	94.1	88.6–99.7	82.04	7
	Molecular methods (combined)	94.1	88.6–99.7	82.04	7
	RDT/PCR	100	98.9–100	ND	3
	Microscopy/RDT/PCR	80.5	68.1–92.8	ND	3
	Microscopy/PCR	100	87.9–100	ND	1
	**Malaysia**	93.6	87.7–99.4	78.37	8
	Molecular methods (combined)	93.6	87.7–99.4	78.37	8
	PCR	99.7	96.4–100	ND	3
	Microscopy/PCR	87.7	74.2–100	87.19	5
	**Cambodia**	91.1	85.9–96.3	98.58	8
	Molecular methods (combined)	95.2	92.4–98.0	94.00	7
	RDT/PCR	98.1	96.6–99.6	ND	2
	Microscopy/RDT/PCR	93.1	84.9–100	ND	3
	PCR	100	99.9–100	ND	2
	Non-molecular methods	69.7	66.6–72.6	ND	1
	Microscopy/RDT	69.7	66.6–72.6	ND	1
	**Indonesia**	90.3	83.6–97.1	88.25	6
	Molecular methods (combined)	90.3	83.6–97.1	88.25	6
	Microscopy/RDT/PCR	98.4	91.4–99.7	ND	1
	Microscopy/PCR	77.2	48.4–90.3	ND	3
	LAMP/PCR	66.7	30.0–90.3	ND	1
	PCR	100	86.7–100	ND	1
	**Thailand**	87.4	72.7–100	96.73	10
	Molecular methods (combined)	89.7	85.7–93.7	28.58	7
	Microscopy/PCR	90.8	82.2–99.5	44.90	4
	Microscopy/LAMP/PCR	89.4	81.1–94.3	ND	1
	RDT/PCR	88.1	83.8–92.5	ND	2
	Non-molecular methods	81.8	40.7–100	ND	3
	Microscopy	81.8	40.7–100	ND	3
	**Vietnam**	69.7	38.4–100	98.48	6
	Molecular methods (combined)	69.7	38.4–100	98.48	6
	Microscopy/PCR	59.4	12.8–100	ND	3
	RDT/PCR	100	93.7–100	ND	1
	PCR	100	61.0–100	ND	1
	Microscopy/RDT/PCR	40.6	31.5–50.3	ND	1
	**Philippines**	23.3	12.3–34.4	ND	2
	Molecular methods (combined)	23.3	12.3–34.4	ND	2
	Microscopy/RDT/PCR	19.6	10.7–33.2	ND	1
	Microscopy/PCR	75.0	30.1–95.4	ND	1
	**Democratic Republic of East Timor** (Microscopy/PCR)	100	74.1–100	ND	1
	**Thailand and Myanmar** (Microscopy/PCR)	100	95.3–100	ND	1
	**Thailand–Myanmar border, Cambodia, Vietnam** (Microscopy/RDT/PCR)	94.9	92.6–96.6	ND	1
	**Thailand–Myanmar border, Cambodia** (PCR)	53.6	51.6–55.6	ND	1
**Western Asia**	**Overall**	74.3	56.4–92.3	97.33	7
	**Molecular methods** (combined)	64.8	30.6–99.0	ND	3
	Microscopy/RDT/PCR	62.7	56.3–69.2	ND	2
	Microscopy/PCR	100	61.0–100	ND	1
	**Non-molecular methods**	81.7	60.8–100	97.44	4
	Microscopy	81.7	60.8–100	97.44	4
	**Iran**	88.2	70.3–100	ND	2
	Molecular methods (combined)	100	61.1–100	ND	1
	Microscopy/PCR	100	61.1–100	ND	1
	Non-molecular methods	70.0	39.7–89.2	ND	1
	Microscopy	70.0	39.7–89.2	ND	1
	**Yemen**	80.8	60.7–100	98.10	4
	Molecular methods (combined)	70.0	62.5–76.6	ND	1
	Microscopy/RDT/PCR	70.0	62.5–76.6	ND	1
	Non-molecular methods	84.4	61.6–100	ND	3
	Microscopy	84.4	61.6–100	ND	3
	**Saudi Arabia** (Microscopy/RDT/PCR)	26.7	14.2–44.4	ND	1
**South Asia**	**Overall**	78.0	70.8–85.1	98.47	20
	**Molecular methods** (combined)	87.2	79.9–94.5	98.10	11
	Microscopy/PCR	74.5	71.2–77.5	ND	1
	Microscopy/RDT/PCR	88.2	81.2–95.2	97.82	9
	RDT/PCR	100	51.0–100	ND	1
	**Non-molecular methods**	66.1	51.9–80.4	97.71	9
	Microscopy	86.3	73.6–99.0	ND	3
	Microscopy/RDT	56.2	32.0–80.4	ND	3
	RDT	6.1	15.8–96.3	ND	3
	**India**	77.4	68.6–86.2	98.65	14
	Molecular methods (combined)	86.0	77.1–95.0	98.14	6
	Microscopy/RDT/PCR	86.0	77.1–95.0	98.14	6
	Non-molecular methods	70.9	56.5–85.2	97.72	8
	RDT	56.1	15.8–96.3	ND	3
	Microscopy	86.3	73.6–99.0	ND	3
	Microscopy/RDT	70.4	63.9–77.0	ND	2
	**Bangladesh**	70.9	53.3–88.5	98.71	4
	Molecular methods (combined)	83.9	66.6–100	ND	3
	Microscopy/PCR	74.5	71.2–77.5	ND	1
	Microscopy/RDT/PCR	92.2	90.0–94.5	ND	2
	Non-molecular methods	25.7	14.2–42.1	ND	1
	Microscopy/RDT	25.7	14.2–42.1	ND	1
	**Pakistan** (Microscopy/RDT/PCR)	100	82.3–100	ND	1
	**Bhutan** (RDT/PCR)	100	51.0–100	ND	1
**Participants**	Participants in communities (all age groups)	85.2	83.3–87.2	98.63	60
	Molecular methods (combined)	87.2	85.3–89.2	98.51	54
	PCR	90.1	68.0–100	99.76	6
	RDT/PCR	92.3	86.7–97.8	92.05	7
	LAMP/PCR	66.7	30.0–90.3	ND	1
	Microscopy/PCR	91.0	87.6–94.4	95.03	19
	Microscopy/RDT/PCR	80.1	76.0–84.2	98.02	20
	Microscopy/LAMP/PCR and restriction enzyme digestion/DNA sequencing	89.4	81.1–94.3	ND	1
	Non-molecular methods	69.2	61.8–76.6	89.28	6
	Microscopy	58.8	51.6–65.9	ND	2
	RDT	78.3	75.5–81.1	ND	2
	Microscopy/RDT	69.8	66.9–72.6	ND	2
	Participants in communities (children)	91.2	84.1–98.4	95.03	8
	Molecular methods (combined)	89.1	78.6–99.6	94.14	5
	RDT/PCR	100	83.9–100	ND	1
	Microscopy/PCR	96.1	91.8–100	ND	2
	PCR	100	86.7–100	ND	1
	Microscopy/RDT/PCR	86.1	83.5–88.3	ND	1
	Non-molecular methods	93.7	80.7–100	ND	3
	Microscopy	93.7	80.7–100	ND	3
	Participants in communities (adults)	97.9	94.2–100	75.32	4
	Molecular methods (combined)	97.5	93.4–100	ND	3
	RDT/PCR	100	97.9–100	ND	1
	Microscopy/RDT/PCR	98.1	95.6–100	ND	2
	Non-molecular methods	100	81.6–100	ND	1
	Microscopy	100	81.6–100	ND	1
	Pregnant women	59.8	31.5–88.1	99.27	6
	Molecular methods (combined)	98.1	95.1–100	ND	2
	Microscopy/RDT/PCR	98.1	95.1–100	ND	2
	Non-molecular methods	42.5	23.3–61.6	96.64	4
	Microscopy	49.1	45.2–52.9	ND	1
	RDT	23.4	17.8–30.2	ND	1
	Microscopy/RDT	58.6	51.1–66.0	ND	2
	Migrant workers	97.8	90.2–100	0	5
	Molecular methods (combined)	100	89.9–100	ND	2
	PCR	100	61.0–100	ND	1
	Microscopy/PCR	100	79.6–100	ND	1
	Non-molecular methods	92.2	74.3–100	ND	3
	Microscopy	92.2	74.3–100	ND	3
	Participants in communities (not specified age group) (Microscopy/RDT/PCR)	100	89.3–100	ND	1
	Participants had undergone training in the forest (Microscopy/PCR)	100	89.3–100	ND	2
	Participants in communities and migrant workers (RDT/PCR)	100	51.0–100	ND	1

*CI* confidence interval, *LAMP* Loop-mediated isothermal amplification, *ND* not determined, *PCR* polymerase chain reaction, *RDT* rapid diagnostic test.

## Discussion

Asymptomatic infection is a disease reservoir that must be considered when determining the malaria risk for malaria-naive individuals and when developing malaria control and elimination strategies in areas transitioning from pre-elimination to elimination, such as in Asia. When the molecular method was used alone or in combination with other methods, the meta-analysis results showed that the prevalence of asymptomatic *Plasmodium* infection in Asia was approximately 6.3% and varied between 5.8% and 6.7%; meanwhile, the proportion of asymptomatic *Plasmodium* infection per all parasitised individuals was high at 84.5% and varied between 79.1% and 89.9%. These results indicate the low prevalence estimates of asymptomatic *Plasmodium* infection in Asian communities where malaria is of low endemicity. The low prevalence estimates of asymptomatic *Plasmodium* infection and a high proportion of asymptomatic cases may be due to the increasing use of highly sensitive methods for the detection of malaria in asymptomatic and submicroscopic individuals. Compared with studies that used molecular methods alone or in combination with other methods, lower pooled prevalence (3.4%) and proportion estimates (72.2%) of asymptomatic *Plasmodium* infection in Asia were documented when non-molecular methods such as microscopy, RDT, or a combination of these two methods were used for the detection of malarial parasites. Consistent with the findings from a previous meta-analysis of the diagnostic accuracy and limited sensitivity of microscopy and RDTs for the detection of asymptomatic *Plasmodium* infections^104^, the pooled results of the present study confirmed that using the molecular method alone or in combination with other methods could increase the chances of detecting asymptomatic *Plasmodium* infection among participants.

In Southeast Asian countries, the subgroup meta-analysis showed the lowest prevalence of asymptomatic *Plasmodium* infections among participants but the participants harbored the highest proportion estimate of asymptomatic *Plasmodium* infections as compared to other areas. The high proportion of asymptomatic *Plasmodium* infection among parasitised individuals in Southeast Asia may be explained by the fact that participants in this area, particularly the adult population, have been exposed to several episodes of *Plasmodium* infection and have acquired immunity against clinical malaria^105^. A previous study suggested that local transmission causes the rapid acquisition of immunity and a reduction in the frequency of symptomatic infections^106^. As asymptomatic *Plasmodium* infections are associated with submicroscopic parasite densities, the proportion of sub-microscopic infections is high, which may indicate that malaria control efforts have been successful and that some populations retain some residual immunity to the infection^107^. Although the prevalence and proportion estimates of asymptomatic *Plasmodium* infection in Southeast Asia were heterogenic, the homogeneity of the proportion estimate of asymptomatic *Plasmodium* infection was found in studies that used molecular methods in combination with non-molecular methods for the detection of asymptomatic *Plasmodium* infection in Thailand^3,27,28,35,64,88,92^. In other parts of Asia, such as Western Asia, there were limited studies that demonstrated asymptomatic *Plasmodium* infection, such as in Yemen, which showed a high prevalence (23%)^24^ and in Iran, which showed a very low prevalence (0.2%) as detected by a combination of molecular and non-molecular methods^59^. This result indicated the heterogeneity of asymptomatic *Plasmodium* infection in these areas. The low prevalence of asymptomatic *Plasmodium* infection in Iran can be explained by the fact that Iran has almost succeeded in eliminating malaria from the country^59^. Meanwhile, the combination of molecular and non-molecular methods detected the high prevalence of asymptomatic *Plasmodium* infection in Yemen as compared to the use of non-molecular methods only^24^. Therefore, this result also indicated an increased sensitivity for the detection of asymptomatic *Plasmodium* infection when molecular methods are used in combination with microscopy or RDT. In South Asia, the prevalence of asymptomatic *Plasmodium* infection was higher in Bangladesh than in India or Pakistan and was the highest in Bhutan. Interestingly, asymptomatic *Plasmodium* infection was reported in areas where malaria was previously almost eliminated, such as Bhutan, due to the transmission of malaria across the Indian border^100,108^.

The systematic review and meta-analysis demonstrated that the highest prevalence of asymptomatic *Plasmodium* infection detected by molecular methods alone or in combination with microscopy or RDT was found among adults. This result indicated that asymptomatic *Plasmodium* infection was more frequently found among adults because members of the adult population in low transmission areas have been exposed to several episodes of *Plasmodium* infection and have acquired immunity against clinical malaria^105^. Age is one of the factors that contribute to asymptomatic *Plasmodium* infection^7,79,109^. Nevertheless, a previous study by van Eijk et al.^7^ showed that young children (5 years old) were much more likely to have sub-microscopic *P. vivax*, which can lead to asymptomatic *P. vivax* infection. The infection with *P. vivax* may result in faster immunity acquisition, and asymptomatic *P.vivax* infection can occur at younger ages than *P. falciparum* infection^109^. The cause of the faster development of clinical immunity to *P. vivax* is unknown; however, frequent population exposure to hypnozoites may be a factor^110^.

The meta-analysis results indicated that using routine diagnostic methods in combination with the highly sensitive method would detect asymptomatic *Plasmodium* infection at a higher rate in this region. The highly sensitive tests for detecting asymptomatic *Plasmodium* infection with low density in monitoring malaria transmission intensity in low transmission areas aid in malaria control and elimination efforts. Although PCR is highly sensitive for the diagnosis of low-density parasitemia, it requires well-trained personnel and expensive laboratory facilities and is not suitable for surveys of asymptomatic *Plasmodium* infection in communities. Additional point-of-care testing is required to determine the true burden of asymptomatic *Plasmodium* infection in low -transmission settings. In Asia, it is possible that more cases of asymptomatic *Plasmodium* infection exist in other areas where malaria surveys have never been conducted, particularly in urban regions of Southeast Asia or China where the prevalence of malaria is low. If the malaria surveys using highly sensitive tests are conducted in those regions, the prevalence estimates of asymptomatic *Plasmodium* infection would be low, but the proportion estimate of asymptomatic *Plasmodium* infection would be high. Although the prevalence estimates of asymptomatic *Plasmodium* infection in Asia were low, particularly in Southeast Asia as compared to other areas of Asia, particularly South Asia, a small prevalence of infections was sufficient to restart malaria transmission, which disturbed malaria elimination goals in several countries. The evidence of the meta-analysis results also implies the need for active case detection in low malaria transmission settings as the transmission of malaria takes place locally. In addition, the aggressive prevention and control of infections by strengthening malaria intervention strategies such as the sufficiently wide distribution of insecticide-treated bed nets (ITN) and the coverage of indoor residual spraying (IRS) are crucially needed. Also, the mass treatment of the asymptomatic infection carriers before the transmission season is important to prevent transmission of infection to reservoirs and to other populations. The sensitive detection, aggressive prevention and control, and also mass treatment of asymptomatic infection reservoirs would be able to reduce the number of infections and disrupt malaria transmission^111^. Furthermore, the findings of the current study will serve as a source of up-to-date information for those who aim to conduct further investigation on asymptomatic *Plasmodium* infection in particular areas of Asia.

The present systematic review has limitations. First, some studies did not show the exact prevalence of asymptomatic *Plasmodium* infection; hence, excluded from the meta-analysis. Second, some studies were excluded because they did not collect clinical information until the patient was positive for malaria by microscopy. Third, the community/participant selection may not have included participants from all age groups, minority ethnicities, and diverse occupations, which may have introduced bias into the asymptomatic *Plasmodium* infection prevalence estimates. Fourth, using only microscopy might cause false negatives, while using only RDT for *Plasmodium* detection might cause false positives, which might underestimate or overestimate the prevalence and proportion estimate of asymptomatic *Plasmodium* infection, respectively. Finally, geographical heterogeneity was a limitation of the study as the frequency of asymptomatic spread varies according to the epidemiological situation, transmission rates, and consequent host immunity.

## Conclusions

Asymptomatic *Plasmodium* infection is a disease reservoir that should be taken into account. There was a low prevalence of asymptomatic *Plasmodium* infection in different parts of Asia, but there was a high proportion of asymptomatic *Plasmodium* infection among all parasitised individuals. These results may support and facilitate the elimination and control programs for asymptomatic *Plasmodium* infection in Asia.

## Supplementary Information

Supplementary Table S1.

Supplementary Table S2.

Supplementary Table S3.

Supplementary Table S4.

## Data Availability

All data relating to the present study are available in this manuscript and supplementary files.
