# Supplementary material for: Prevalence and proportion estimate of asymptomatic Plasmodium infection in Asia: a systematic review and meta-analysis
Source: Sci Rep. 2023 Jun 27;13:10379. doi: 10.1038/s41598-023-37439-9 (PMC10300031; doi:10.1038/s41598-023-37439-9)
Supplement: Supplementary file 1 — Supplementary Table S1. [file 41598_2023_37439_MOESM1_ESM.docx]

**Table S1. Search strategy**

**Embase**

**16 October 2022**

| No. | Query | Results |
| --- | --- | --- |
| #4 | #1 AND #2 AND #3 | 207 |
| #3 | asia OR asian | 544,600 |
| #2 | asymptomatic OR inapparent OR subclinical OR presymptomatic | 356,835 |
| #1 | 'malaria'/exp OR malaria OR 'plasmodium'/exp OR plasmodium OR 'remittent fever' OR 'marsh fever'/exp OR 'marsh fever' OR 'paludism'/exp | 159,475 |

**PubMed**

**16 October 2022**

| Search number | Query | Search Details | Results |
| --- | --- | --- | --- |
| 4 | #1 AND #2 AND #3 | ("asymptomatic"[All Fields] OR "asymptomatically"[All Fields] OR "asymptomatics"[All Fields]) AND ("malaria"[MeSH Terms] OR "malaria"[All Fields] OR "malarias"[All Fields] OR "malaria s"[All Fields] OR "malariae"[All Fields] OR "malaria"[MeSH Terms] OR ("plasmodium"[MeSH Terms] OR "plasmodium"[All Fields] OR "plasmodiums"[All Fields] OR "plasmodium s"[All Fields]) OR "plasmodium"[MeSH Terms]) AND ("asia"[MeSH Terms] OR "asia"[All Fields] OR "asia"[MeSH Terms] OR ("asians"[MeSH Terms] OR "asians"[All Fields] OR "asian"[All Fields]) OR "asians"[MeSH Terms]) | 369 |
| 3 | ((((Asia) OR (Asia[MeSH Terms]))) OR (Asian)) OR (Asian[MeSH Terms]) | "asia"[MeSH Terms] OR "asia"[All Fields] OR "asia"[MeSH Terms] OR "asians"[MeSH Terms] OR "asians"[All Fields] OR "asian"[All Fields] OR "asians"[MeSH Terms] | 1,207,394 |
| 2 | (((malaria) OR (malaria[MeSH Terms])) OR (Plasmodium)) OR (Plasmodium[MeSH Terms]) | "malaria"[MeSH Terms] OR "malaria"[All Fields] OR "malarias"[All Fields] OR "malaria s"[All Fields] OR "malariae"[All Fields] OR "malaria"[MeSH Terms] OR "plasmodium"[MeSH Terms] OR "plasmodium"[All Fields] OR "plasmodiums"[All Fields] OR "plasmodium s"[All Fields] OR "plasmodium"[MeSH Terms] | 119,311 |
| 1 | (asymptomatic[MeSH Terms]) OR (asymptomatic) | "asymptomatic"[All Fields] OR "asymptomatically"[All Fields] OR "asymptomatics"[All Fields] | 185,328 |

**Ovid**

| **Search terms/Search strategy** | **Date** |
| --- | --- |
| (malaria OR Plasmodium OR “remittent fever” OR “marsh fever” OR paludism) AND (asymptomatic OR inapparent OR subclinical OR presymptomatic) AND (Asia OR Asian)  **Filter:** limit 4 to (ovid full text available and articles with abstracts and original articles)  **Results: 85** | 16 October 2022 |

**MEDLINE**

| **Search terms/Search strategy** | **Date** |
| --- | --- |
| (malaria OR Plasmodium OR “remittent fever” OR “marsh fever” OR paludism) AND (asymptomatic OR inapparent OR subclinical OR presymptomatic) AND (Asia OR Asian)  **Filter:** None  **Results: 124** | 16 October 2022 |

**Scopus**

16 October 2022

| Search number | **Search terms/Search strategy** | **Results** |
| --- | --- | --- |
| 4. | ( TITLE-ABS-KEY ( malaria OR plasmodium OR "remittent fever" OR "marsh fever" OR paludism ) ) AND ( TITLE-ABS-KEY ( asymptomatic OR inapparent OR subclinical OR presymptomatic ) ) AND ( TITLE-ABS-KEY ( asia OR asian ) ) | 131 |
| 3. | TITLE-ABS-KEY ( asia OR asian ) | 667,626 |
| 2. | TITLE-ABS-KEY ( asymptomatic OR inapparent OR subclinical OR presymptomatic ) | 278,857 |
| 1. | TITLE-ABS-KEY ( malaria OR plasmodium OR "remittent fever" OR "marsh fever" OR paludism ) | 152,121 |
