# Supplementary material for: Prevalence and proportion estimate of asymptomatic Plasmodium infection in Asia: a systematic review and meta-analysis
Source: Sci Rep. 2023 Jun 27;13:10379. doi: 10.1038/s41598-023-37439-9 (PMC10300031; doi:10.1038/s41598-023-37439-9)
Supplement: Supplementary file 4 — Supplementary Table S4. [file 41598_2023_37439_MOESM4_ESM.docx]

**Table S4. Meta-regression results**

| **Meta-analysis** | **Covariates** | **P value** | **I-squared residual** |
| --- | --- | --- | --- |
| **Prevalence estimate of asymptomatic malaria among participants** | Publication years | 0.625 | 99.37 |
|  | Part of Asian continent | 0.84 | 99.38 |
|  | Participants ‘group | 0.40 | 99.38 |
|  | Age group | 0.72 | 99.34 |
|  | Method for malaria detection | 0.78 | 99.32 |
| **Proportion estimate of asymptomatic malaria among all malaria-positive cases** | Publication years | 0.714 | 99.86 |
|  | Part of Asian continent | 0.06 | 99.71 |
|  | Participants ‘group | < 0.01 | 98.46 |
|  | Age group | 0.59 | 99.72 |
|  | Method for malaria detection | 0.33 | 99.76 |
